# Economy of Labour in Hospital Management

**Published:** 1912-09-14

**Authors:** W. Fenwick

**Affiliations:** Assistant Secretary, Hull Royal Infirmary. Hull.


### The Editor's Letter-Box.

### Economy of Labour in Hospital Management.

*To the Editor of* The Hospital.

Sir,—I shall be obliged if you will allow me space for reply to Mr. A. M. Hooper, who, writing under the above heading, offers criticism respecting a printed form mentioned in my short article of 27th ult., in connection with which the words "Daily Report" should have preceded paragraph in question. I regret that instead of explaining more fully I only devoted five lines to the same and as a hint to your readers. To remove doubt as to the value of this "Daily Report" I beg to add that both the patients' names and numbers are shown opposite the condition as staged—*e.g.*, "Dangerously ill, friends may visit," "For operation to-day," etc., etc. All other patients not calling for special remark are grouped under the condition "Comfortable," irrespective of name or number. Any change in a patient's condition is reported and the necessary alterations made in list, one of which is issued by each ward daily.

I thank Mr. Hooper for drawing attention to this matter, and would assure readers that this report answers admirably.

The abuse of telephone is a subject that has recently been discussed at this hospital, and I am afraid that the time it would take a telephone operator to explain to inquirers otherwise than the nearest (?) relatives that such calls could not be answered would much exceed that required for reply as to patients' condition.—Yours respectfully, W. Fenwick,

Assistant Secretary, Hull Royal Infirmary.

Hull.

